# Supplementary material for: Iterative improvement in the automatic modular design of robot swarms
Source: PeerJ Comput Sci. 2020 Dec 7;6:e322. doi: 10.7717/peerj-cs.322 (PMC7924708; doi:10.7717/peerj-cs.322)
Supplement: Supplemental Information 3 [file peerj-cs-06-322-s003.zip › argos3/doc/api/standalone/a00488_source.html]

ARGoS: plugins/robots/generic/control\_interface/ci\_colored\_blob\_omnidirectional\_camera\_sensor.cpp Source File


- Main Page
- Related Pages
- Namespaces
- Classes
- Files

- File List
- File Members

# plugins/robots/generic/control\_interface/ci\_colored\_blob\_omnidirectional\_camera\_sensor.cpp

Go to the documentation of this file.

```
00001 #include "ci_colored_blob_omnidirectional_camera_sensor.h"
00002 
00003 #ifdef ARGOS_WITH_LUA
00004 #include <argos3/core/wrappers/lua/lua_utility.h>
00005 #endif
00006 
00007 namespace argos {
00008 
00009    /****************************************/
00010    /****************************************/
00011 
00012 #ifdef ARGOS_WITH_LUA
00013    int LuaEnableOmnidirectionalCamera(lua_State* pt_lua_state) {
00014       /* Perform action */
00015       CLuaUtility::GetDeviceInstance<CCI_ColoredBlobOmnidirectionalCameraSensor>(pt_lua_state, "colored_blob_omnidirectional_camera")->Enable();
00016       return 0;
00017    }
00018 #endif
00019 
00020    /****************************************/
00021    /****************************************/
00022 
00023 #ifdef ARGOS_WITH_LUA
00024    int LuaDisableOmnidirectionalCamera(lua_State* pt_lua_state) {
00025       /* Perform action */
00026       CLuaUtility::GetDeviceInstance<CCI_ColoredBlobOmnidirectionalCameraSensor>(pt_lua_state, "colored_blob_omnidirectional_camera")->Disable();
00027       return 0;
00028    }
00029 #endif
00030 
00031    /****************************************/
00032    /****************************************/
00033 
00034 #ifdef ARGOS_WITH_LUA
00035    void CCI_ColoredBlobOmnidirectionalCameraSensor::CreateLuaState(lua_State* pt_lua_state) {
00036       CLuaUtility::OpenRobotStateTable(pt_lua_state, "colored_blob_omnidirectional_camera");
00037       CLuaUtility::AddToTable(pt_lua_state, "_instance", this);
00038       CLuaUtility::AddToTable(pt_lua_state, "enable", &LuaEnableOmnidirectionalCamera);
00039       CLuaUtility::AddToTable(pt_lua_state, "disable", &LuaDisableOmnidirectionalCamera);
00040       for(size_t i = 0; i < m_sReadings.BlobList.size(); ++i) {
00041          SBlob& sBlob = *(m_sReadings.BlobList[i]);
00042          CLuaUtility::StartTable(pt_lua_state, i+1);
00043          CLuaUtility::AddToTable(pt_lua_state, "distance", sBlob.Distance);
00044          CLuaUtility::AddToTable(pt_lua_state, "angle", sBlob.Angle);
00045          CLuaUtility::AddToTable(pt_lua_state, "color", sBlob.Color);
00046          CLuaUtility::EndTable(pt_lua_state);
00047       }
00048       CLuaUtility::CloseRobotStateTable(pt_lua_state);
00049    }
00050 #endif
00051 
00052    /****************************************/
00053    /****************************************/
00054 
00055 #ifdef ARGOS_WITH_LUA
00056    void CCI_ColoredBlobOmnidirectionalCameraSensor::ReadingsToLuaState(lua_State* pt_lua_state) {
00057       lua_getfield(pt_lua_state, -1, "colored_blob_omnidirectional_camera");
00058       /* Save the number of elements in the blob list */
00059       size_t unLastBlobNum = lua_rawlen(pt_lua_state, -1);
00060       /* Overwrite the table with the new messages */
00061       for(size_t i = 0; i < m_sReadings.BlobList.size(); ++i) {
00062          SBlob& sBlob = *(m_sReadings.BlobList[i]);
00063          CLuaUtility::StartTable(pt_lua_state, i+1);
00064          CLuaUtility::AddToTable(pt_lua_state, "distance", sBlob.Distance);
00065          CLuaUtility::AddToTable(pt_lua_state, "angle", sBlob.Angle);
00066          CLuaUtility::AddToTable(pt_lua_state, "color", sBlob.Color);
00067          CLuaUtility::EndTable(pt_lua_state);
00068       }
00069       /* Are the new messages less than the old ones? */
00070       if(m_sReadings.BlobList.size() < unLastBlobNum) {
00071          /* Yes, set to nil all the extra entries */
00072          for(size_t i = m_sReadings.BlobList.size()+1; i <= unLastBlobNum; ++i) {
00073             lua_pushnumber(pt_lua_state,  i);
00074             lua_pushnil   (pt_lua_state    );
00075             lua_settable  (pt_lua_state, -3);
00076          }
00077       }
00078       lua_pop(pt_lua_state, 1);
00079    }
00080 #endif
00081 
00082    /****************************************/
00083    /****************************************/
00084 
00085    const CCI_ColoredBlobOmnidirectionalCameraSensor::SReadings& CCI_ColoredBlobOmnidirectionalCameraSensor::GetReadings() const {
00086       return m_sReadings;
00087    }
00088 
00089 }
```

---

Generated on 10 Jul 2018 for ARGoS by 
 1.6.1
